# Documentation of overweight or obesity diagnosis codes during adult primary care encounters

**DOI:** 10.1016/j.obpill.2026.100320

**Published:** 2026-08-12

**Authors:** Cameron I. Bradfield, Michael G. Heckman, Sheikh Fahad, Dawn M. Mussallem, Michael J. Maniaci, Kaitlin M. Moran

**Affiliations:** a Wake Forest University School of Medicine, 475 Vine Street, Winston-Salem, NC, 27101, United States; b Department of Medical Education, Mayo Clinic, 4500 San Pablo Road, Jacksonville, FL, 32224, United States; c Division of Clinical Trials and Biostatistics, Mayo Clinic, 4500 San Pablo Road, Jacksonville, FL, 32224, United States; d Department of Integrative Medicine, Mayo Clinic, 4500 San Pablo Road, Jacksonville, FL, 32224, United States; e Department of Hospital Internal Medicine, Mayo Clinic, 4500 San Pablo Road, Jacksonville, FL, 32224, United States; f Robert and Monica Jacoby Center for Breast Health, Mayo Clinic, 4500 San Pablo Road, Jacksonville, FL, 32224, United States

**Keywords:** Body mass index, Diagnosis codes, Obesity, Overweight, Primary care, Retrospective study

## Abstract

**Background:**

Obesity is highly prevalent among U.S. adults and is associated with substantial morbidity and mortality. Primary care clinicians are well positioned to identify patients with overweight or obesity and address related health risks. Prior studies suggest that obesity is often underdiagnosed, but less is known about how frequently overweight or obesity diagnosis codes are documented during routine primary care encounters.

**Methods:**

This was a retrospective observational study of 49,279 adult family medicine and internal medicine encounters at Mayo Clinic in Jacksonville, Florida, from July 1, 2021, through June 30, 2022. Encounters included adults ≥18 years of age with a recorded body mass index (BMI) ≥25 kg/m^2^. The primary outcome was documentation of an overweight or obesity diagnosis code (OODC) during the encounter. Logistic regression evaluated associations between patient, clinician, and encounter characteristics and OODC documentation.

**Results:**

An OODC was documented in 3658 encounters (7.4%). Documentation increased across BMI categories, from 2.5% of encounters involving patients with overweight to 22.0% of encounters involving patients with class III obesity. In multivariable analysis, class III obesity had the strongest association with OODC documentation compared with overweight (adjusted odds ratio [aOR], 11.22; 95% confidence interval [CI], 10.05, 12.55). Male clinician sex was associated with modestly lower odds of documentation (aOR, 0.88; 95% CI, 0.81, 0.95). Internal medicine encounters had higher odds of documentation than family medicine encounters (aOR, 1.24; 95% CI, 1.14, 1.35), while office visits had lower odds than comprehensive visits (aOR, 0.70; 95% CI, 0.65, 0.75).

**Conclusion:**

OODCs were infrequently documented during adult primary care encounters involving patients with BMI ≥25 kg/m^2^. Documentation increased with BMI severity but remained uncommon overall and varied according to clinician age, clinician sex, department, and encounter type.

## Graphical abstract

**Figure:**
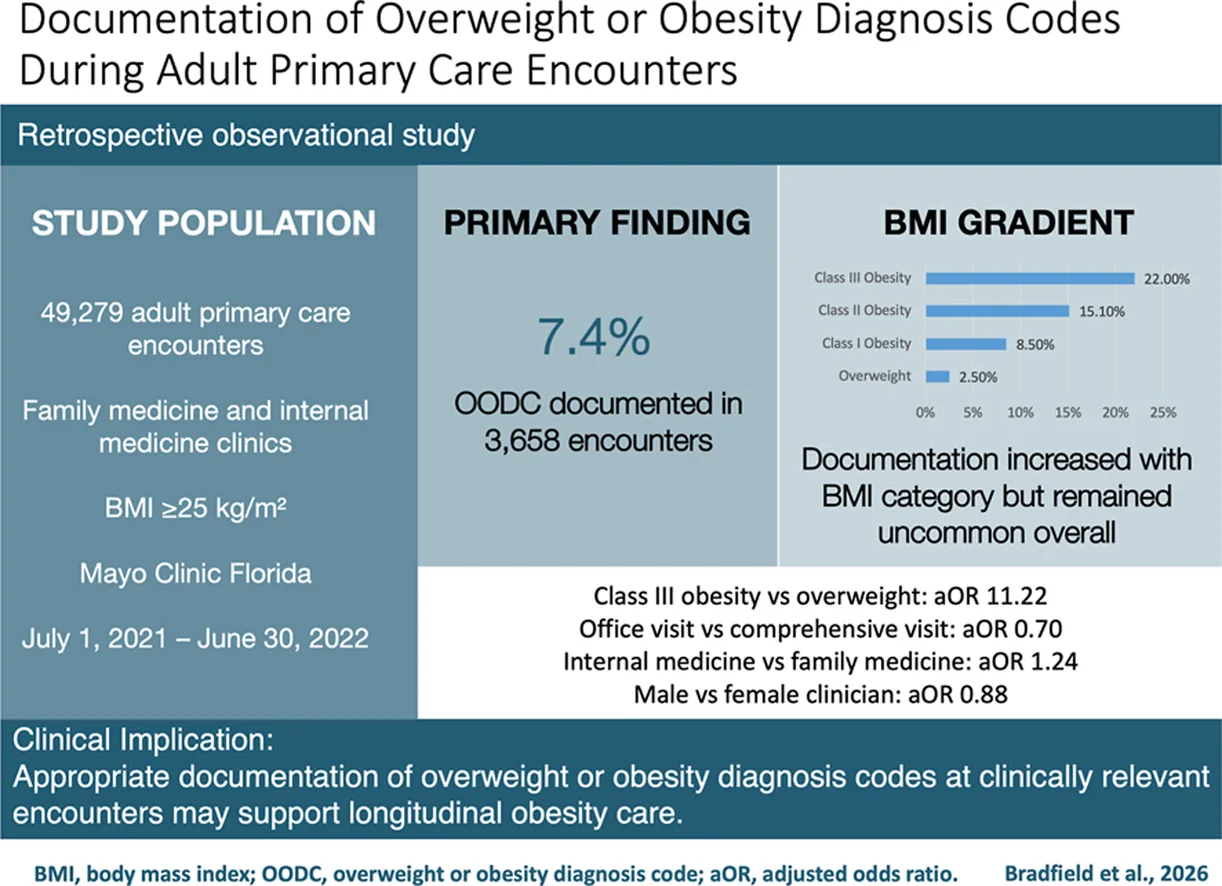

## 1 Introduction

Overweight and obesity are highly prevalent chronic health conditions encountered routinely in adult primary care and are associated with substantial morbidity and mortality [[1], [2], [3], [4]]. Primary care clinicians are often positioned to identify elevated body mass index (BMI), address weight-related health risks, and support longitudinal follow-up for patients with overweight or obesity.

Prior studies have shown that obesity is often underdiagnosed in clinical practice [[5], [6], [7]]. This gap is clinically important because documentation of overweight or obesity may influence problem list visibility, future follow-up, counseling, referral, and treatment consideration. At the same time, diagnosis documentation does not occur uniformly across encounters. Prior work suggests that outpatient documentation of sensitive or preventive health issues may vary according to patient characteristics, clinician characteristics, specialty, and visit structure [[8], [9], [10], [11]]. These factors may be especially relevant in primary care, where clinicians often balance preventive care, chronic disease management, acute concerns, medication management, and competing visit priorities. Accordingly, we selected patient age and sex; clinician age, sex, department, and resident status; encounter type; and patient-clinician pairing characteristics because prior literature suggests that documentation practices and clinical decision making may vary according to patient, clinician, and visit-level factors.

Despite prior work on obesity underdiagnosis, less is known about encounter-level documentation of overweight or obesity diagnosis codes (OODCs) during routine adult primary care visits among patients who already meet BMI criteria for overweight or obesity. It is also unclear whether OODC documentation varies according to BMI category, clinician department, clinician sex, resident status, encounter type, or patient-clinician pairing characteristics. Understanding these patterns may help identify clinical contexts in which weight-related diagnosis code documentation is less consistent.

The purpose of this retrospective observational study was to evaluate OODC documentation among adult family medicine and internal medicine encounters involving patients with BMI ≥25 kg/m^2^. Specifically, we sought to determine how frequently OODCs were documented during eligible primary care encounters and to identify patient, clinician, and encounter characteristics associated with OODC documentation. We hypothesized that OODC documentation would be uncommon overall, would increase with BMI category, and would vary according to clinician and encounter characteristics.

## 2 Methods

### 2.1 Study design

This was a retrospective observational study of adult primary care encounters in the departments of family medicine and internal medicine at Mayo Clinic in Jacksonville, Florida. The study period extended from July 1, 2021, through June 30, 2022. Data were extracted from the electronic health record by Mayo Clinic Data Provisioning Services. This was a single-site study conducted in the United States. The study did not involve prospective assignment to an intervention and did not require clinical trial registration.

The study was reviewed by the Mayo Clinic Institutional Review Board and was determined to be exempt from the requirement for IRB [Application #: 22-013497] because of the retrospective design and use of existing clinical data.

To create a more homogeneous sample of comparable in-person primary care encounters, the following encounter types were excluded before analysis: ancillary procedure, documentation, education, erroneous encounter, nurse only, procedure visit, and telemedicine. Encounters with missing values for variables used in the regression analyses were excluded by complete case analysis.

### 2.2 Study population

The unit of analysis was the clinical encounter. We identified all family medicine and internal medicine visits during the study period and then restricted the cohort to encounters involving adults ≥18 years of age with a recorded body mass index (BMI) ≥25 kg/m^2^ at the time of the visit. Because the unit of analysis was the encounter, individual patients could contribute more than 1 encounter during the study period. Clinician demographic and practice characteristics were obtained from the electronic health record and associated institutional data sources.

### 2.3 Outcome definition

The primary outcome was documentation of an overweight or obesity diagnosis code (OODC) during the encounter. An OODC was operationally defined as a visit diagnosis indicating overweight or obesity, including International Classification of Diseases, Tenth Revision codes E66.0 through E66.9, or a BMI-specific diagnosis code indicating BMI ≥25 kg/m^2^, including codes Z68.25 through Z68.45. Because direct measurement of counseling, shared decision-making, treatment, or referral was not available, the presence of 1 of these diagnosis codes was used as a marker of coded documentation of overweight or obesity during the visit.

### 2.4 Study variables

Study variables were selected based on availability in the electronic health record and prior literature suggesting that diagnosis documentation and clinical decision making may vary according to patient, clinician, and encounter characteristics. Patient characteristics included age, sex, and BMI. BMI was analyzed both as a continuous variable and as a categorical variable. BMI categories were defined according to standard clinical thresholds as 25.0–29.9 kg/m^2^ for overweight, 30.0–34.9 kg/m^2^ for class I obesity, 35.0–39.9 kg/m^2^ for class II obesity, and ≥40.0 kg/m^2^ for class III obesity.

Clinician characteristics included age, sex, department affiliation, and resident status. Department affiliation was classified as family medicine or internal medicine. Resident status was classified as resident or attending physician. Patient and clinician sex were obtained from the electronic health record and institutional data fields and were categorized as male or female. Gender identity was not available for analysis.

Encounter characteristics included visit type, categorized as comprehensive visit or office visit. Comprehensive visits were defined as visits with longer scheduled time intended to address preventive care or multiple chronic conditions, whereas office visits were defined as shorter routine encounters typically focused on a more limited set of concerns. Encounter type was determined from the visit classification recorded in the electronic health record scheduling system.

We also evaluated paired patient-clinician characteristics, including patient-clinician sex combination and patient-clinician age combination. Sex combinations were categorized as male patient, male clinician; male patient, female clinician; female patient, male clinician; and female patient, female clinician. Age combination was categorized by grouping patient age as ≤54 years or >54 years and clinician age as ≤44 years or >44 years.

### 2.5 Statistical analysis

This was a retrospective observational analysis of encounter-level data. Continuous variables were summarized as medians with ranges, and categorical variables were summarized as frequencies and percentages. Associations between patient, clinician, and encounter characteristics and OODC documentation were evaluated with logistic regression models. Results are reported as odds ratios (ORs) with 95% confidence intervals (CIs).

Each characteristic was first examined in a model adjusted for BMI category. Multivariable models were then adjusted for BMI category, clinician department, encounter type, and patient-clinician age combination, except when a given variable was the predictor of interest, in which case that variable was omitted from the adjustment set. Patient age and clinician age were associated with OODC documentation in BMI-adjusted analyses but were not included as simultaneous adjustment covariates because of collinearity with patient-clinician age combination. After Bonferroni correction for the 10 characteristics evaluated, two-sided *P* values < 0.005 were considered statistically significant. Because this was a retrospective observational analysis, estimates of association and 95% CIs were emphasized in interpretation. The available analysis used encounter-level logistic regression and did not include random effects or cluster-robust standard errors. All analyses were performed with R statistical software, version 4.1.2.

## 3 Results

### 3.1 Cohort characteristics

A total of 49,279 adult family medicine and internal medicine encounters met inclusion criteria. An OODC was documented in 3658 encounters, representing 7.4% of the study cohort.

The median patient age was 60 years, and 23,670 encounters (48.0%) involved male patients. The median BMI was 31.1 kg/m^2^. Encounters involving patients with overweight accounted for 24,390 encounters (49.5%), while 14,850 (30.1%) involved patients with class I obesity, 6069 (12.3%) involved patients with class II obesity, and 3970 (8.1%) involved patients with class III obesity.

The median clinician age was 45 years, and 21,141 encounters (42.9%) involved male clinicians. Most encounters occurred in family medicine rather than internal medicine, and 7684 encounters (15.6%) involved resident physicians. Office visits accounted for 32,534 encounters (66.0%), while 16,745 encounters (34.0%) were comprehensive visits. Patient, clinician, encounter, and pairing characteristics are summarized in Table 1.

**Table 1:** Patient, clinician, and encounter characteristics among adult primary care encounters involving patients with BMI ≥25 kg/m.^2^.

Characteristic	N	Value
**Patient characteristics**		
Age, years	49,279	60 (18, 102)
Age category, years	49,279	
≤34		4066 (8.3)
35 to 44		5470 (11.1)
45 to 54		8501 (17.3)
55 to 64		13,554 (27.5)
≥65		17,688 (35.9)
Sex recorded as male	49,279	23,670 (48.0)
BMI, kg/m^2^	49,279	31.1 (25.0, 81.0)
BMI category, kg/m^2^	49,279	
25.0 to 29.9, overweight		24,390 (49.5)
30.0 to 34.9, class I obesity		14,850 (30.1)
35.0 to 39.9, class II obesity		6069 (12.3)
≥40.0, class III obesity		3970 (8.1)
Overweight or obesity diagnosis code documented during encounter	49,279	3658 (7.4)
**Clinician characteristics**		
Age, years	49,279	45 (28, 69)
Age category, years	49,279	
≤34		10,006 (20.3)
35 to 44		14,508 (29.4)
45 to 54		15,094 (30.6)
55 to 64		6431 (13.1)
≥65		3240 (6.6)
Sex recorded as male	49,279	21,141 (42.9)
Department	49,279	
Family medicine		39,583 (80.3)
Internal medicine		9696 (19.7)
Resident physician	49,279	7684 (15.6)
**Encounter characteristics**		
Encounter type	49,279	
Comprehensive visit		16,745 (34.0)
Office visit		32,534 (66.0)
**Patient-clinician pairing characteristics**		
Patient-clinician sex combination	49,279	
Male patient, male clinician		12,859 (26.1)
Male patient, female clinician		10,811 (21.9)
Female patient, male clinician		8282 (16.8)
Female patient, female clinician		17,327 (35.2)
Patient-clinician age combination	49,279	
Patient ≤54 years, clinician ≤44 years		9839 (20.0)
Patient ≤54 years, clinician >44 years		8198 (16.6)
Patient >54 years, clinician ≤44 years		14,675 (29.8)
Patient >54 years, clinician >44 years		16,567 (33.6)

BMI, body mass index. Values are median (minimum, maximum) or n (%), unless otherwise indicated. The unit of analysis was the clinical encounter. Percentages may not total 100 because of rounding.

### 3.2 Documentation of OODCs

OODC documentation was uncommon overall and increased across BMI categories. An OODC was documented in 614 encounters (2.5%) involving patients with overweight, 1256 encounters (8.5%) involving patients with class I obesity, 916 encounters (15.1%) involving patients with class II obesity, and 872 encounters (22.0%) involving patients with class III obesity.

In BMI-adjusted analyses, department, encounter type, clinician age, and patient-clinician age combination were associated with OODC documentation. Patient sex, clinician sex, resident physician status, and patient-clinician sex combination were not associated with OODC documentation in BMI-adjusted analyses using the prespecified Bonferroni-corrected threshold.

### 3.3 Multivariable associations with OODC documentation

In full multivariable analyses, BMI category remained strongly associated with OODC documentation. Compared with encounters involving patients with overweight, the odds of OODC documentation were higher for encounters involving patients with class I obesity (adjusted odds ratio [aOR], 3.62; 95% confidence interval [CI], 3.28, 4.00), class II obesity (aOR, 7.02; 95% CI, 6.31, 7.82), and class III obesity (aOR, 11.22; 95% CI, 10.05, 12.55).

Several clinician and encounter characteristics were also associated with OODC documentation. Clinician age was associated with documentation overall (*P* < 0.001); compared with clinicians aged ≤34 years, clinicians aged ≥65 years had higher odds of documentation (aOR, 1.45; 95% CI, 1.25, 1.68; *P* < 0.001). Male clinician sex was associated with modestly lower odds of documentation compared with female clinician sex (aOR, 0.88; 95% CI, 0.81, 0.95; *P* < 0.001). Internal medicine encounters had higher odds of OODC documentation than family medicine encounters (aOR, 1.24; 95% CI, 1.14, 1.35; *P* < 0.001), while office visits had lower odds than comprehensive visits (aOR, 0.70; 95% CI, 0.65, 0.75; *P* < 0.001). Patient-clinician age combination was also associated with OODC documentation overall (*P* < 0.001), although none of the individual contrasts met the prespecified Bonferroni-corrected threshold.

**Fig. 1:**
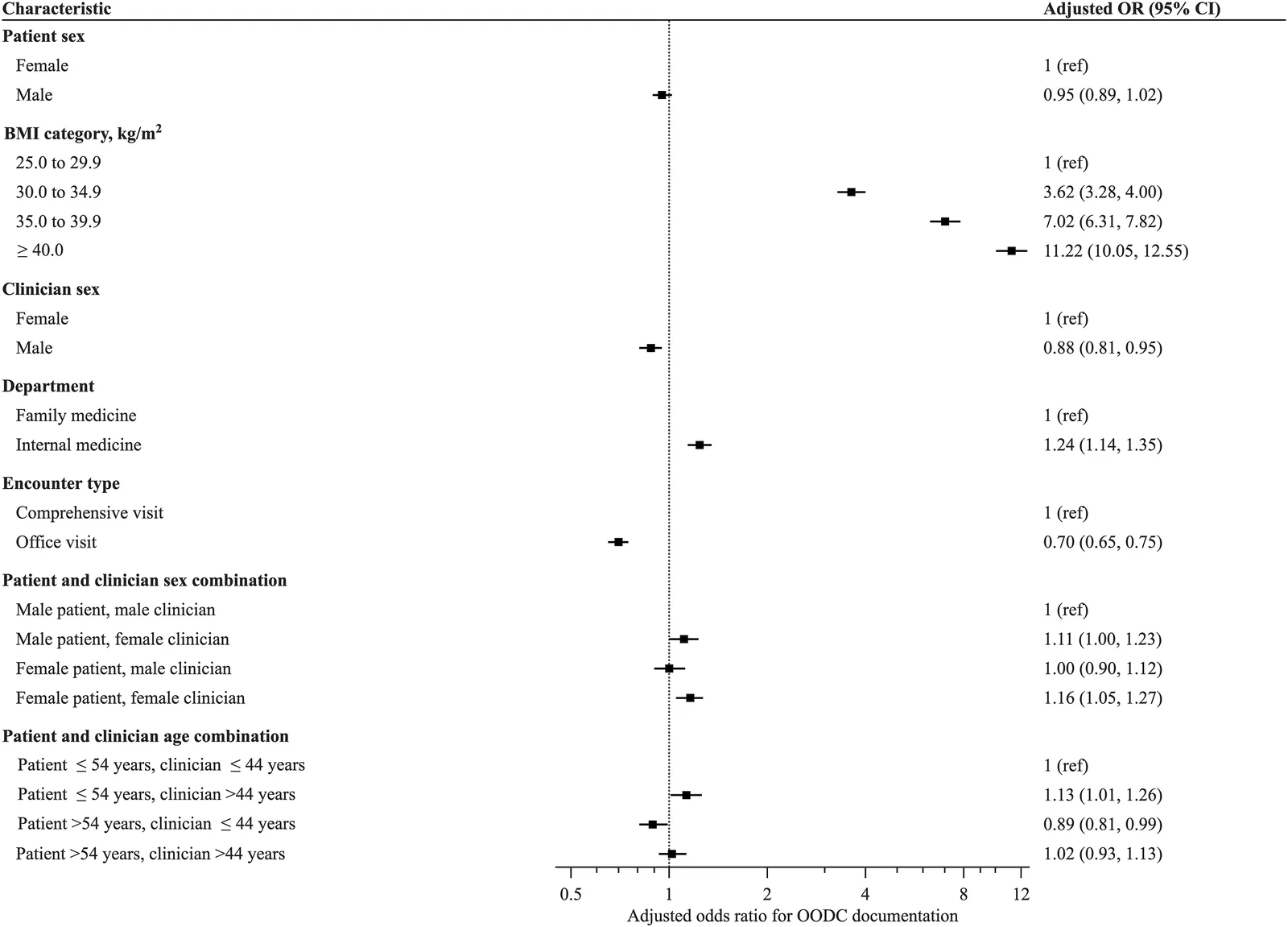
Full multivariable associations of selected patient, clinician, and encounter characteristics with overweight or obesity diagnosis code (OODC) documentation. Forest plot showing adjusted odds ratios and 95% confidence intervals for OODC documentation during adult primary care encounters involving patients with BMI ≥25 kg/m^2^. Odds ratios >1 indicate higher odds of documentation, and odds ratios <1 indicate lower odds of documentation. Reference groups are labeled as ref. BMI, body mass index; CI, confidence interval; OODC, overweight or obesity diagnosis code; OR, odds ratio.

The patient and clinician sex combination did not meet the prespecified Bonferroni-corrected threshold overall (*P* = 0.007). Because the omnibus test did not reach statistical significance, formal evaluation of individual contrasts was deferred. Exploratory review of the subgroup pairings showed an adjusted odds ratio of 1.16 (95% CI, 1.05, 1.27; *P* = 0.003) for encounters involving female patients and female clinicians compared to the reference group. Full regression results are shown in Table 2, and selected full multivariable associations are shown in Fig. 1.

**Table 2:** Associations of patient, clinician, and encounter characteristics with overweight or obesity diagnosis code documentation.

Characteristic	N	OODC documented, n (%)	BMI-adjusted OR (95% CI)	*P* value	Full multivariable OR (95% CI)	*P* value
**Patient characteristics**						
Age category, years				Overall *P* = 0.030		Overall *P* = 0.022
≤34	4066	314 (7.7)	1.00 (reference)		1.00 (reference)	
35 to 44	5470	441 (8.1)	1.00 (0.85, 1.16)	0.96	0.99 (0.85, 1.16)	0.94
45 to 54	8501	793 (9.3)	1.12 (0.98, 1.29)	0.10	1.12 (0.97, 1.29)	0.12
55 to 64	13,554	990 (7.3)	0.96 (0.84, 1.11)	0.61	0.95 (0.83, 1.09)	0.46
≥65	17,688	1120 (6.3)	0.97 (0.85, 1.11)	0.69	0.97 (0.84, 1.11)	0.63
Patient sex						
Female	25,609	2018 (7.9)	1.00 (reference)		1.00 (reference)	
Male	23,670	1640 (6.9)	0.98 (0.91, 1.05)	0.55	0.95 (0.89, 1.02)	0.18
BMI category, kg/m^2^				Overall *P* < 0.001		Overall *P* < 0.001
25.0 to 29.9	24,390	614 (2.5)	1.00 (reference)		1.00 (reference)	
30.0 to 34.9	14,850	1256 (8.5)	3.58 (3.24, 3.95)	<0.001	3.62 (3.28, 4.00)	<0.001
35.0 to 39.9	6069	916 (15.1)	6.88 (6.19, 7.66)	<0.001	7.02 (6.31, 7.82)	<0.001
≥40.0	3970	872 (22.0)	10.90 (9.77, 12.17)	<0.001	11.22 (10.05, 12.55)	<0.001
**Clinician characteristics**						
Age category, years				Overall *P* < 0.001		Overall *P* < 0.001
≤34	10,006	720 (7.2)	1.00 (reference)		1.00 (reference)	
35 to 44	14,508	1048 (7.2)	1.01 (0.91, 1.12)	0.88	1.02 (0.92, 1.13)	0.69
45 to 54	15,094	1154 (7.6)	1.12 (1.01, 1.24)	0.027	1.14 (1.03, 1.26)	0.012
55 to 64	6431	451 (7.0)	1.02 (0.90, 1.16)	0.74	1.02 (0.90, 1.16)	0.73
≥65	3240	285 (8.8)	1.41 (1.22, 1.64)	<0.001	1.45 (1.25, 1.68)	<0.001
Clinician sex						
Female	28,138	2155 (7.7)	1.00 (reference)		1.00 (reference)	
Male	21,141	1503 (7.1)	0.96 (0.89, 1.03)	0.21	0.88 (0.81, 0.95)	<0.001
Department						
Family medicine	39,583	2897 (7.3)	1.00 (reference)		1.00 (reference)	
Internal medicine	9696	761 (7.8)	1.21 (1.11, 1.31)	<0.001	1.24 (1.14, 1.35)	<0.001
Resident physician						
No	41,595	3083 (7.4)	1.00 (reference)		1.00 (reference)	
Yes	7684	575 (7.5)	1.00 (0.91, 1.10)	0.99	1.06 (0.95, 1.19)	0.28
**Encounter characteristics**						
Encounter type						
Comprehensive visit	16,745	1468 (8.8)	1.00 (reference)		1.00 (reference)	
Office visit	32,534	2190 (6.7)	0.69 (0.64, 0.74)	<0.001	0.70 (0.65, 0.75)	<0.001
**Patient-clinician pairing characteristics**						
Patient-clinician sex combination				Overall *P* = 0.26		Overall *P* = 0.007
Male patient, male clinician	12,859	891 (6.9)	1.00 (reference)		1.00 (reference)	
Male patient, female clinician	10,811	749 (6.9)	0.99 (0.90, 1.10)	0.90	1.11 (1.00, 1.23)	0.061
Female patient, male clinician	8282	612 (7.4)	0.96 (0.86, 1.07)	0.44	1.00 (0.90, 1.12)	0.98
Female patient, female clinician	17,327	1406 (8.1)	1.05 (0.96, 1.15)	0.31	1.16 (1.05, 1.27)	0.003
Patient-clinician age combination				Overall *P* < 0.001		Overall *P* < 0.001
Patient ≤54 years, clinician ≤44 years	9839	817 (8.3)	1.00 (reference)		1.00 (reference)	
Patient ≤54 years, clinician >44 years	8198	731 (8.9)	1.11 (1.00, 1.24)	0.053	1.13 (1.01, 1.26)	0.026
Patient >54 years, clinician ≤44 years	14,675	951 (6.5)	0.90 (0.81, 0.99)	0.030	0.89 (0.81, 0.99)	0.028
Patient >54 years, clinician >44 years	16,567	1159 (7.0)	1.02 (0.93, 1.13)	0.63	1.02 (0.93, 1.13)	0.65

BMI, body mass index; CI, confidence interval; OODC, overweight or obesity diagnosis code; OR, odds ratio. ORs, 95% CIs, and *P* values were estimated using logistic regression models. BMI-adjusted models were adjusted for BMI category, except when BMI category was the predictor of interest. Full multivariable models were adjusted for BMI category and variables associated with OODC documentation at *P* < 0.05 in BMI-adjusted analyses. These variables were department, encounter type, and patient-clinician age combination. When one of these variables was the predictor of interest, it was omitted from the adjustment set. Patient age and clinician age were also associated with OODC documentation at *P* < 0.05 in BMI-adjusted analyses; however, because of collinearity with patient-clinician age combination, they were not included as simultaneous adjustment covariates. Two-sided *P* values < 0.005 were considered statistically significant after Bonferroni correction for multiple testing. Percentages may not total 100 because of rounding.

## 4 Discussion

This retrospective observational study evaluated how often OODCs were documented during adult primary care encounters involving patients with BMI ≥25 kg/m^2^ and whether documentation varied by patient, clinician, and encounter characteristics. OODC documentation occurred in only 7.4% of eligible encounters. Documentation increased progressively with BMI category but remained uncommon overall, including among encounters involving patients with obesity. Documentation also varied by clinician age, clinician sex, department, and encounter type. The pairing of patient and clinician sex was not statistically significant overall at the prespecified Bonferroni-corrected threshold (overall *P* = 0.007). An isolated exploratory finding suggested higher documentation odds in encounters with female patients and female clinicians, but this observation requires validation in independent cohorts. These findings extend prior work on obesity underdiagnosis by focusing specifically on encounter-level diagnosis code documentation during routine adult primary care visits.

These results are consistent with prior studies showing that obesity is frequently underdiagnosed in routine clinical practice [[5], [6], [7]]. The present findings add to that literature by showing that OODC documentation was uncommon across a large cohort of adult family medicine and internal medicine encounters, even though all included encounters involved patients who met BMI criteria for overweight or obesity. The gradient across BMI categories is clinically important. Documentation was lowest among encounters involving patients with overweight and increased progressively among patients with class I, class II, and class III obesity. This pattern suggests that diagnosis code documentation may occur more consistently as BMI increases. However, patients with overweight or lower severity obesity may represent an important group in whom early risk identification and longitudinal follow-up could be particularly relevant.

The association between encounter type and OODC documentation may reflect the structure and competing demands of primary care visits. Office visits had lower odds of OODC documentation than comprehensive visits. This finding is plausible because shorter, problem-focused visits may leave less room for preventive care, chronic disease recognition, and documentation of conditions not directly related to the presenting concern. In this context, low documentation may not reflect lack of clinician awareness alone. It may also reflect visit priorities, coding workflows, time constraints, or uncertainty about whether a weight-related diagnosis should be added during a specific encounter.

The associations involving clinician age, clinician sex, department, and patient-clinician sex pairing should be interpreted cautiously. Clinician age was associated with OODC documentation overall, with higher odds of documentation among clinicians aged ≥65 years compared with clinicians aged ≤34 years. Male clinician sex was associated with modestly lower odds of OODC documentation, and internal medicine encounters had higher odds of documentation than family medicine encounters. While an exploratory signal suggested higher documentation odds in encounters with female patients and female clinicians, the overarching sex combination variable did not achieve statistical significance. This study was not designed to determine why these patterns occurred. Potential explanations include differences in coding habits, visit content, communication patterns, comfort discussing weight, preventive care emphasis, or patient case mix not fully captured in the available data. These findings are best interpreted as evidence that diagnosis documentation may vary across clinical context and clinician characteristics, rather than as evidence of specific clinician behavior.

A central implication of this study is the distinction between documentation and clinical action. The outcome in this analysis was documentation of an OODC during the encounter, not counseling, shared decision-making, referral, pharmacotherapy, nutrition therapy, physical activity counseling, behavioral intervention, or bariatric surgery referral. It is therefore possible that weight was discussed or addressed in some encounters without being coded as a visit diagnosis. Conversely, diagnosis code documentation does not prove that counseling or treatment occurred. Even with that distinction, documentation remains clinically meaningful. Diagnosis codes may influence problem list visibility, future follow-up, referral patterns, insurance coverage for obesity-related services, and whether elevated BMI is revisited longitudinally.

These findings are especially relevant as obesity care continues to evolve. Evidence-based obesity management increasingly includes structured lifestyle intervention, obesity medications, bariatric procedures, and longitudinal risk management. Within that context, inconsistent OODC documentation may represent one barrier to systematic obesity care. Improving documentation alone would not be sufficient to improve outcomes, and these findings should not be interpreted as supporting routine addition of an OODC at every eligible encounter. Rather, appropriate documentation at clinically relevant encounters may help support longitudinal recognition and follow-up of overweight and obesity as part of broader obesity care. Future work should evaluate whether diagnosis code documentation is associated with subsequent counseling, referral, obesity medication use, bariatric surgery referral, or longitudinal weight-related follow-up. Additional studies should also determine whether changes to electronic health record prompts, problem list workflows, or visit templates improve documentation without increasing stigma or unnecessary administrative burden.

## 5 Limitations

Several limitations should be considered when interpreting these findings. First, because this was a retrospective observational study, the analysis is subject to residual confounding and misclassification. The observed associations identify patterns in diagnosis code documentation, but they do not establish why those patterns occurred.

Second, the presence of an OODC was used as a marker of coded documentation of overweight or obesity during the encounter. This measure does not capture the full content of the clinical visit. Weight may have been discussed, counseled, monitored, or addressed without being coded as a visit diagnosis. Conversely, diagnosis code documentation does not confirm that counseling, referral, pharmacotherapy, or other obesity-related management occurred. Clinicians may also have recognized elevated BMI through prior problem list documentation, note text, or longitudinal follow-up without assigning an OODC at the index encounter.

Third, because the unit of analysis was the clinical encounter, the observed documentation frequency should not be interpreted to mean that an overweight or obesity diagnosis code was clinically indicated at every eligible visit. Some encounters may have focused on concerns unrelated to weight, and repeated documentation of an OODC at encounters in which weight was not being addressed may be unnecessary. In addition, patients who review visit diagnoses and other electronic health record information may perceive repeated inclusion of a weight-related diagnosis at unrelated encounters as an inappropriate emphasis on their weight, which could contribute to weight-related stigma or adversely affect the patient-clinician relationship. The present study did not assess the reason for each encounter, patient preferences regarding weight-related documentation, patient perceptions of diagnosis coding, or effects of such documentation on the therapeutic relationship.

Fourth, the study cohort was defined using BMI, which is a practical and widely used screening measure but is not a direct measure of body fat, its distribution, or adiposity-related risk. BMI may not fully characterize obesity-related risk across individuals and does not account for differences related to fat distribution, metabolic health, comorbid conditions, age, sex, or ethnicity [12].

Fifth, the study was conducted within a single health system, which may limit generalizability to other practice settings with different patient populations, coding practices, visit structures, documentation workflows, or obesity care resources.

Sixth, the unit of analysis was the clinical encounter rather than the individual patient. Patients with multiple eligible visits may have contributed more than 1 observation to the dataset. In addition, encounters were nested within clinicians, and the available analysis did not account for clustering at the patient or clinician level. Because the models did not account for within-patient or within-clinician correlation, standard errors may be underestimated, and confidence intervals and *P* values for clinician and encounter characteristics may be overly precise. Therefore, associations involving clinician and encounter characteristics should be interpreted cautiously.

Seventh, the number and pattern of otherwise eligible encounters excluded because of missing covariate data were not available in the completed analytic dataset, which limits interpretation of potential selection bias from complete case analysis.

Despite these limitations, this study provides a large encounter-level assessment of OODC documentation in adult primary care. The findings identify clinically relevant patterns in documentation across BMI category, encounter type, clinician characteristics, and patient-clinician pairing characteristics. These results may help inform future work evaluating documentation workflows and longitudinal obesity care in primary care settings.

## 6 Conclusion

In this retrospective observational study of adult primary care encounters involving patients with BMI ≥25 kg/m^2^, OODCs were documented in only 7.4% of eligible encounters. Documentation increased with BMI severity but remained uncommon overall, including among encounters involving patients with obesity. These findings indicate that encounter-level coded documentation of overweight and obesity was infrequent and varied according to patient weight status and clinical context.

Improving obesity care in primary care may require systems that support appropriate recognition and documentation of overweight and obesity at clinically relevant encounters while facilitating longitudinal follow-up of weight-related health risks. Because this study evaluated diagnosis code documentation rather than counseling, referral, or treatment, future work should examine how documentation patterns relate to actual obesity care delivery, patient perceptions, and clinical outcomes.

**Takeaway clinical messages**

• Overweight or obesity diagnosis codes were documented in a small minority of adult primary care encounters involving patients with BMI ≥25 kg/m^2^.
• Documentation increased with BMI category, suggesting that patients with overweight or lower severity obesity may be especially likely to lack diagnosis code documentation.
• Appropriate documentation of overweight and obesity at clinically relevant encounters may support longitudinal recognition and follow-up while avoiding unnecessary repetitive coding.

## Credit author statement

Kaitlin M. Moran: Conceptualization; Methodology; Supervision

Cameron I. Bradfield: Writing original draft; Writing review and editing; Formal analysis; Data curation; Visualization.

Michael G. Heckman: Formal analysis; Software.

Sheikh Fahad: Formal analysis.

Dawn M. Mussallem: Conceptualization.

Michael J. Maniaci: Conceptualization.

All authors reviewed and approved the final manuscript.

## Ethical adherence and ethical review

This retrospective observational study was approved by the Mayo Clinic Institutional Review Board. The requirement for informed consent was waived because the study used existing clinical data. This study did not prospectively assign participants to an intervention and did not require clinical trial registration.

## Data availability

The data underlying this study are not publicly available because they contain protected health information and are subject to institutional privacy restrictions.

## Declaration of artificial intelligence and AI-assisted technologies

During preparation of this manuscript, generative AI technology was used to assist with figure code generation and language editing. After using this tool, the authors reviewed and edited the content as needed and take full responsibility for the content of the publication.

## Source of funding

This research did not receive any specific grant from funding agencies in the public, commercial, or not-for-profit sectors. Internal Mayo Clinic resources supported the conduct of the study.

## Declaration of competing interests

None of the authors have any financial declarations or conflicts of interest to report.
